# Knowledge, Attitudes, and Practices Regarding Menstrual Hygiene among Girls in Ghizer, Gilgit, Pakistan

**DOI:** 10.3390/ijerph20146424

**Published:** 2023-07-21

**Authors:** Sanober Fazal Shah, Neelam Saleem Punjani, Syeda Naghma Rizvi, Sana Sadiq Sheikh, Rafat Jan

**Affiliations:** 1 School of Nursing and Midwifery, Aga Khan University, Karachi 75950, Pakistan; sanober.fazal21@alumni.aku.edu (S.F.S.); naghma.rizvi@aku.edu (S.N.R.); rafat.jan@aku.edu (R.J.); 2 Faculty of Nursing, University of Alberta, Edmonton, AB T6G 1C9, Canada; 3 Tabba Heart Institute, Karachi 75950, Pakistan; sana.sheikh2@aku.edu

**Keywords:** menstrual hygiene, menstruation, knowledge, attitudes, practices, adolescents

## Abstract

Introduction: Menstrual hygiene is a critical issue encountered by women and girls of reproductive age that negatively affects their health and empowerment. It is still deemed a taboo subject in several parts of the world, and girls hesitate to discuss menstruation with their family members, friends, or schoolteachers, which creates hurdles when they experience their menarche. Girls residing in rural areas encounter more problems, since they lack proper resources and knowledge to manage their menstruation in school as well as at home. The purpose of this study was to evaluate the knowledge, attitudes, and practices regarding the menstrual hygiene of girls residing in rural areas of Gilgit, Pakistan. Methods: A descriptive cross-sectional design was used to assess the study questions. The consecutive sampling technique was applied to recruit 300 female participants from remote areas of Gilgit, Pakistan, who were 13 to 22 years old. A pre-tested questionnaire was utilized to collect the data, and SPSS version 21.0 was used to analyze them. Results: The study found that more than half of the participants had a poor level of knowledge and practices and negative attitudes towards menstrual hygiene. This could be due to many cultural and social factors associated with menstrual hygiene. Conclusions: The study concluded that the study participants were not knowledgeable about menstrual hygiene. They had poor practices and attitudes regarding menstrual hygiene. Hence, it is recommended that frequent sessions should be conducted by healthcare workers to enhance the knowledge of parents, teachers, and young girls, to increase their positive attitudes and practices regarding menstrual hygiene.

## 1. Introduction

According to the World Health Organization (WHO), sexual and reproductive health issues are a rising problem around the globe. Among them, menstrual hygiene is a critical issue encountered by women and girls of reproductive age that negatively affects their health and empowerment [1]. Across the globe, 1.8 billion girls menstruate each month, yet a major portion of this population lacks adequate knowledge as well as basic facilities to handle their menstruation in an appropriate and healthy way [2]. Moreover, menstruation is still deemed a taboo subject in several parts of the world [3,4]. The literature states that young people in low- and middle-income countries (LMICs) confront challenges related to menstruation and menstrual hygiene practices due to religious, cultural, and social constraints and due to incorrect information [5]. Moreover, girls residing in rural areas encounter more problems, since they lack the proper resources, skills, and knowledge to manage their menstruation in school as well as at home [6]. In Pakistan, menstruating girls have insufficient information about practices regarding menstruation and menstrual hygiene and, consequently, this impacts the wellbeing of such girls [7].

The WHO has defined adolescents as those aged 10 to 19 years, whereas young people range from 10 to 24 years of age. Adolescence and adulthood are crucial as such individuals experience major changes in their physical, hormonal, emotional, behavioral, and mental health. Young people are more likely to adopt unhealthy behaviors and encounter many problems related to their sexual and reproductive health [8]. Because of this transition, young girls face difficulties in managing their menstruation and are exposed to societal taboos [9] However, menstruation or menses is a normal process in females when they reach reproductive age [10].There is evidence that, both in developed and underdeveloped countries, girls experience the early onset of menstruation due to changes in lifestyle, environmental factors, and geographical location [11]. A study in Pakistan reported an age of 12 to 14 years for menarche [11]. The literature highlights that a lack of appropriate information at the first menstrual cycle creates many problems for girls in managing their menstrual hygiene [3,12]

The WHO and UNICEF’s joint monitoring program (JMP) has defined menstrual hygiene management as the usage of hygienic material for menstrual management, to absorb the blood of menstruating girls or women every month [13]. This can be changed as per need in an environment that provides privacy, respect, and comfort, and permits the washing of the hands and body, when required, with soap and clean water, as well as the ability to discard of the used materials properly. Improper menstrual hygiene management (MHM) can lead to low self-esteem, negative body image, and unsafe sexual practices [14]. Considering its impact on women’s lives, May 28th is celebrated as “Global Menstrual Hygiene Day”, which was introduced by WASH United to create awareness and to recognize the rights of girls to manage their menstruation hygienically [15].

Girls in developing countries encounter problems in managing menstruation properly because they lack the basic facilities of WASH (water, sanitation, and hygiene), proper information, a suitable environment, and support, which ultimately, affects their basic human rights to education, health, and privacy [16,17]. This is due to a lack of knowledge, improper awareness, and poor attitudes and practices towards menstruation and menstrual hygiene [5,9]. Additionally, several studies have revealed that the lack of availability of WASH facilities at schools, anxiety about blood dripping onto clothes, a lack of access to sanitary pads, and the poor attitudes of male students and teachers towards menstruation are linked with poor menstrual hygiene [18]. Moreover, girls residing in rural areas, and studying in lower classes, have demonstrated poor knowledge of menstruation [17,18] These factors ultimately affect the health of girls, thus becoming an issue of public health [17].

Moreover, another article has identified that insufficient knowledge and awareness about menstruation builds harmful practices and, thus, it can cause pelvic infections, cervical cancer, school dropouts, poor academic outcomes, and low standards of living [19]. The literature also states that more than 500 million females lack access to basic services for menstrual hygiene management, which is why they suffer from substantial challenges in maintaining menstrual hygiene in a safe and respectable manner [6]. This evidence indicates that there is a need to break the silence on menstruation and empower girls and women by educating them and enabling them to achieve their full potential. Thus, this descriptive study sought to evaluate the knowledge, attitudes, and practices (KAP) regarding menstrual hygiene of girls residing in rural areas in Gilgit, Pakistan.

## 2. Materials and Methods

This study employed a quantitative research design using the cross-sectional descriptive approach. It was conducted at two selected government educational institutions in the district of Ghizer. Ghizer is one of the 10 districts of Gilgit Baltistan, Pakistan. Punyal is the tehsil of Ghizer. The weather in this district is very cold; during the winter season, the temperature falls to 08 degrees Fahrenheit. As per the 2017 census, the total population of Ghizer is 170,000 [20]. Gahkuch is 72 km away from the main city, Gilgit, and it is the capital city of Ghizer. According to UNICEF Pakistan, the majority of the population of Ghizer is in the fourth (34%) quintile [21]. This indicates the poor socioeconomic status of the area. The reason for selecting this region was its convenient location, opportunities, and the availability of many students from different locations, cultures, and socioeconomic backgrounds. Moreover, the only female college in the territory of Punyal is located here. Hence, this allowed the researchers to collect data from a diverse group of students, within a limited timeframe.

### 2.1. Sampling Strategy and Sample Size

Consecutive sampling was applied to collect data from the participants. The sample size was determined using the Open Epi software version 3.01, with a significance level of 95% and a 5% margin of error. The total population of students from 7th grade to 12th grade was 625. The anticipated frequency taken from the previous literature was 50% [11]. The sample size after calculation was 239 participants, and when a 10% attrition rate was added, the sample size obtained was 265 girls. A total of 300 participants were included in the study.

### 2.2. Study Population

The participants who were selected for the collection of data were female students from 7th to 12th grade who had reached the age of menarche (the age limit was 13 to 24 years), who were willing to participate in the study and were residents of Ghizer, Gilgit, Pakistan. Moreover, they could easily understand and write in English or Urdu. However, participants who were not prepared to participate in the study and those who did not sign, or their guardian did not sign, the consent form were excluded from the study. To conduct the aforementioned study, approval was obtained from the Ethical Review Committee (ERC) of Aga Khan University, Pakistan. Participants were assured about their privacy, confidentiality, and anonymity throughout the study.

### 2.3. Data Collection Procedure

For the collection of data, a standardized questionnaire was used. The questionnaire to evaluate the knowledge, attitudes, and practices was taken from a study that was conducted and used in the context of Sindh, Pakistan [22]. However, a few modifications were made in the questions according to the study context, after obtaining permission from the primary author. The tool considered the demographic details of the participants and their knowledge, attitudes, and practices regarding menstrual hygiene. Moreover, multiple responses and single responses were included in the questionnaire. The study questionnaires were translated into the national language, Urdu, by Urdu language experts. Additionally, to eliminate any discrepancies, and to maintain the exact meaning and quality of the questionnaires, back-translation was performed by an independent expert who was not aware of the previous translation in both languages. A pilot study was carried out with 10% of the chosen sample, to rule out any inappropriateness and discrepancies in filling the questionnaire. The questionnaires were distributed and collected by the researchers.

Official permission was obtained from the education directors of Gilgit and the principals of the respective school and college. Eligible participants were asked to give their consent. The participants were thoroughly briefed about the purpose of the study, the risks and benefits, their voluntary participation, and their right to withdraw from the study whenever they wished. Likewise, parents/guardians were approached through the eligible participants and consent was also obtained from them. Furthermore, each guardian or parent’s consent form was checked by the researchers for their signatures and then an assent form was provided to the adolescents. The data collection was completed in three months, from May 2021 to July 2021.

### 2.4. Data Entry and Data Analysis

After the collection of data, the researchers checked the completed questionnaires for their completeness and accuracy. The data were than analyzed through the Statistical Package for Social Sciences (SPSS) version 21. The mean and standard deviation were calculated for continuous variables and frequencies and percentages for categorical variables. The frequencies and percentages have been presented via tables.

## 3. Results

### 3.1. Demographic Details of the Study Participants

In total, 300 young girls participated in the study, and the mean age (±standard deviation) of the study participants was 17.5 (±1.72) years, ranging from 13 to 22 years. The analysis of their fathers’ education showed that 33% (n = 99) had no formal education, whereas, regarding their mothers’ education, 65.3% (n = 196) had no education. Furthermore, participants were also asked if they had attended any class/orientation on menstrual hygiene, and the results showed that only 44.3% (n = 133) had been to an orientation/class on menstrual hygiene, and the provider of the orientation/class was a healthcare provider in 34% (n = 102) and a schoolteacher in 10.3% (n = 31) of cases. The demographic details are provided in Table 1.

### 3.2. Knowledge of Young Girls Regarding Menstrual Hygiene

It was interesting to observe that at the age of menarche, 51.7% (n = 155) were not aware of menstruation, and, among the participants who were aware, namely 47% (n = 141), their main source of information was their mother, representing 41.8% (n = 59). Most young girls, 48.3% (n = 145), mentioned that they had discussed their first periods with their mothers. With regard to the source of menstrual blood, very few participants, 32.3% (n = 97), knew that menstrual blood originated from the uterus: meanwhile, 59% (n = 177) did not know the answer. Moreover, regarding knowledge about menstrual absorbents, around 30% (n = 146) and 33.9% (n = 165) of the participants had heard about disposable pads and reusable cloths/towels, respectively. In addition, 45.7% (n = 137) of the respondents knew that women of reproductive age experienced menstruation, while 33% (n = 99) believed that only adolescent girls experienced menstruation. Almost an equal number of participants, 29% (n = 89) and 28.3% (n = 87), felt that menstruation was a sign of fertility and uncleanliness, respectively. When asked about the start of menstruation, around 68.8% (n = 207) correctly identified that it starts during adolescence. Table 2 shows the frequency distribution of the participants’ knowledge of menstrual hygiene.

### 3.3. Attitudes of Young Girls Regarding Menstrual Hygiene

The second aim of the study was to identify the attitudes of the target population. The analysis showed that around 37.6% (n = 141) of participants mentioned that washing the body during menstruation should be avoided, and 17.1% (n = 64) mentioned avoiding exercise. The majority of the girls, 38.2% (n = 137), were comfortable talking about or asking for advice on menstruation from their mothers, and 29% (n = 104) mentioned their sisters. Approximately 67.3% (n = 202) stated that they experienced anxiety and stress at their menarche, and, to solve this issue, the majority, namely 68.3% (n = 205), felt that their mothers/sisters/friends/relatives should tell them in advance. Additionally, 91% (n = 273) agreed that girls should be made aware before their first cycle, and approximately 42.8% (n = 238) stated that their mothers/sisters/friends/relatives should tell them. Table 3 shows the attitudes of young girls regarding menstrual hygiene.

### 3.4. Practices of Young Girls Regarding Menstrual Hygiene

Most of the study participants, 47.7% (n = 143), used reusable cloths/towels as menstrual absorbents, and 30.7% (n = 92) used disposable pads. Among those who used disposable pads, 24.7% (n = 74) burned them and only 6.3% (n = 19) of them used a dustbin. Among the respondents who used reusable absorbents, the majority of them washed them with soap and water; to dry the washed absorbent, 39.5% (n = 68) chose to dry it in the sun outside, whereas 29.1% (n = 50) hid it under other clothes. A major proportion of the participants, 90.1% (n = 263), reported that they stored the cleaned material in a closed area. During menstruation, nearly half of the young girls, 49.3% (n = 148), changed their absorbents twice a day, while 23% (n = 69) changed them once a day. Regarding the cleaning of the genitals and the area around the genitals, nearly equal numbers of participants, 38.7% (n = 116), mentioned using only water, and 38% (n = 114) mentioned using soap and water. Moreover, more than half, 59.3% (n = 178), reported bathing/washing their bodies when their periods were over. The majority of the girls avoided activities during menstruation, and the analysis showed that around 28.5% (n = 105) avoided washing the body, followed by those who avoided working outside home, at 20.1% (n = 74). Table 4 summarizes the frequency distribution of menstrual hygiene practices.

## 4. Discussion

A study in Nepal mentioned that 47.5% participants had heard about menstruation when they were at the age of 10–12 years [23], while, in the current study, more than half (51.3%) had heard about menstruation when they were 12–15 years. The current study found that all the participants had toilet facilities and soap at home, and 69.75% had soap at school/college; however, the availability of toilets was 100%. Nevertheless, this is not always the case. Other studies in Asian countries, including Pakistan [24,25] and Ethiopia [26], have found that schools have no proper toilet and sanitation facilities.

In 2019, UNICEF recommended including menstrual hygiene management in the curricula of primary schools, in the Asian and African region, so that young girls can learn about reproductive health and menstruation prior to their menarche [27]. However, this study found that more than half of the participants had not received education regarding menstrual hygiene at school/college. This could be due to a lack of attention towards menstrual hygiene in schools and colleges, as no topics are included regarding menstrual hygiene at the school level and teachers ignore such topics. A study in Quetta, Pakistan, reported that nearly 80% of the respondents had no prior education on menstrual hygiene [28]. A systematic review and meta-analysis in India concluded that there was little menstrual hygiene education in schools and this leads to the development of many misconceptions and poor practices regarding menstrual hygiene [29].

### 4.1. Knowledge of Young Girls Regarding Menstrual Hygiene

The results of this study revealed that the participants had a poor level of knowledge. This finding is contradicted by other studies carried out in Indonesia, with 66.6% [30], Nepal, with 87.7% [31], and Afghanistan, with 53.3% [32], where participants had good level of knowledge regarding menstrual hygiene. This could be due to the absence of proper sexual and reproductive health education at the school level, and the majority of the mothers (65.3%) being uneducated in the current study area. This was a hurdle in providing correct knowledge to young girls [33].

Moreover, it was also evident in the current study that a significant number of the participants were not aware of menstruation when they experienced their first periods. This finding is in contrast with a study performed in China, where the majority, 78.1%, of the participants already had knowledge of menstruation because their mothers had informed them about it [34]. However, results similar to those of the current study were also reported in India [35] and in Pakistan [36], where 51.7% and 82.5% of the participants, respectively, had no prior information about menstruation when they experienced their menarche. This can also be linked with social taboos and cultural influences in LMICs, where menstruation is still thought of as a shameful event and females are reluctant to discuss it openly [37]. This is why prior information about and awareness of menstrual hygiene is important.

The majority of the respondents (41.8%) in the present study mentioned their mothers as their main source of information regarding menstruation, followed by their sisters (31.6%). This may be because menstruation is a feminine process and girls feel more comfortable discussing it with their mothers and sisters. These findings are also reflected in a systematic review of LMICs, including Pakistan [5]. Moreover, the current study found that 48.3% of girls discussed their first menstruation with their mothers. A study in Ghana also documented that 61.8% of girls preferred to discuss the menarche with their mothers [38]. However, many studies have concluded that mothers and sisters in LMICs possess incorrect information about menstruation and this poor knowledge is transferred to young girls, which negatively affects their levels of knowledge and practices regarding menstrual hygiene [5,39].

Additionally, this study found that 20% of the participants were not aware of what to do at the time of menarche, and this could be because most of the girls were not aware of menstruation before the age of menarche. Almost similar results were mentioned in a study conducted in Ethiopia, South Sudan, Uganda, Tanzania, and Zimbabwe, where almost 66% of girls had no knowledge about the management of menstruation [40]. The root cause of these negative experiences may be the cultural stigma and the social context around menstruation, which is common worldwide [41]. 

It is very surprising that almost 59% of the respondents were unaware of the source of menstrual blood, and few of them correctly identified that menstrual blood originates from the uterus. This may be due to inappropriate information regarding menstruation obtained from the participants’ mothers/sisters, who were not educated, or because the participants had not attended any educational sessions on menstrual hygiene in their school/college. These results are aligned with other studies [11,28,42]. Almost equal proportions of girls in the study had heard about menstrual absorbents, which included disposable pads (30%) and reusable cloths/towels (33.9%). A similar study was conducted in Gambia, where approximately 97% of girls had knowledge about reusable cloths, while 87% were aware of disposable pads [43].

This study revealed that the majority of the girls (45.7%) knew that women of reproductive age experience menstruation, while 19% did not know. This can be explained by the fact that some students at the intermediate level had attended awareness sessions on menstrual hygiene, so they might have obtained the information from these. This was a unique finding in our study, as no other study was found that had assessed this type of knowledge. In the current study, despite having poor knowledge regarding menstruation, it was found that participants were mostly aware that menstruation was a natural process. Other studies in Pakistan, Ghana, Indonesia, Nigeria, Ethiopia, Bangladesh, and Nepal also found that a high number of girls knew that menstruation was a normal physiological process [12,30,44].

Another interesting finding was that nearly an equal number of participants reported menstruation as a sign of fertility (29%) and uncleanliness (28.3%), while 21.2% believed that it was sign of being ready to marry. The current study also found that only 4.7% of the girls were married. The possible reasons for the aforementioned findings, in the context of Gilgit, are that when girls start menstruating, they are considered to be mature enough to be married and bear children. However, studies from the United States, Ghana, and Pakistan found different results, where the majority of the girls reported that menstruation was a sign of puberty and adulthood [11,39,45]. Moreover, a major proportion of the sample in present study mentioned that menstruation started during adolescence. This is in line with other studies from Bhutan and Pakistan [12,46]. 

### 4.2. Attitudes of Young Girls Regarding Menstrual Hygiene

The findings of the current study showed both negative and positive attitudes regarding menstrual hygiene. This can be explained by the fact that many of the young girls had no prior information about menstruation when they experienced it for the first time, and, in rural areas, menstruation is associated with many taboos and the girls had grown up in such an environment. For example, they believe that girls become unclean when they are menstruating and they are not allowed to cook or wash utensils. These results are in line with other studies performed in Saudi Arabia and Nepal, where girls had negative attitudes regarding menstrual hygiene [23,39].

The majority of the participants stated that girls should not take baths or wash their bodies when menstruating, followed by some stating that they should not work outside the home. A possible reason for this could be that in Pakistani society, menstruation is thought to be a dirty process by many, and taking a bath during menstruation is believed to lead to the swelling of the body and to cause more bleeding [47]. Similar findings were reported in studies from Saudi Arabia and India [48,49]. Furthermore, this study found that many of the girls could comfortably talk about menstruation with their mothers and sisters, while approximately 9.7% preferred not to discuss it with anyone. This is because girls in Pakistani society are more connected to their mothers and sisters. Moreover, it is not considered appropriate in this region to discuss feminine topics with anyone else, such as male members of the family, as menstruation is considered a very sensitive topic to discuss. These findings are contradicted in other studies of LMICs [5,21]. 

This study further found that the girls were aware of their diet during menstruation; however, myths were strongly present, as many of them mentioned avoiding sweets and spicy food, while a few of them mentioned avoiding cold water. The findings about the avoidance of sweets were unique to this study. This could have been because of the belief in the study area that sweets cause more bleeding; meanwhile, many studies have mentioned the avoidance of cold water and sour and spicy foods [47,50]. Furthermore, the present study concluded that more than half of the participants experienced stress and anxiety at the onset of their menarche. A possible explanation for this could be a lack of prior awareness and guidance from parents and educational institutions regarding menstruation and its management, which ultimately creates anxiety, stress, and negative attitudes. The literature also shows similar findings, where girls mention stress, shame, and anxiety during menarche [3,51,52].

Moreover, a large number of girls felt that they should be made aware before menarche, and 42.8% of the girls suggested that their mothers, sisters, friends, relatives, or peers should tell them, while only 1.1% mentioned schoolteachers. This indicates that girls need more and accurate information on this natural phenomenon, which can help them to manage their menstrual hygiene without any problems. The possible reasons for the low percentage of schoolteachers could be that, in Gilgit, there is no concept of sexual and reproductive health in schools/colleges, and teachers are not trained to provide education in this area. They avoid discussions of such topics, although teachers could provide accurate information about menstrual hygiene. A scoping review of LMICs also reported that girls should be informed about menarche by their mothers, sisters, female friends, or teachers [53].

### 4.3. Practices of Young Girls Regarding Menstrual Hygiene

The current study found that the majority of the participants were using reusable cloths/towels as menstrual absorbents, and a lesser proportion of participants (30.7%) were using disposable pads. This could be due to financial issues, less awareness, the lower availability of disposable pads, hesitation in purchasing them from the market, and issues of disposal. These findings are not consistent with other studies, including those conducted in Pakistan, where the majority of girls were found to use disposable pads ([12,36,54,55,56]). Moreover, of those who used disposable materials, most of them burned them. The reason is that, in Gilgit, there are no proper waste management systems or garbage collectors, and girls are expected to burn their used pads at night.

This study also found that some good practices were followed by many of the participants, such as the use of soap and water to wash the reusable cloths/towels and drying them in the sun. A possible reason for this could be the wide availability of water and soap in schools and at home. These findings concur with other studies [36,42,43,57]. However, one study from Pakistan contradicts the current study’s findings, as it found that the majority of girls dried their reusable pads in dark and hidden places [47]. The present study also found that nearly all young girls stored the clean material in closed areas (box/drawer/closet/clean bag). The findings of a few other studies reflect those of the current study [58,59].

The literature has mentioned that a greater proportion of girls change their soaked pads twice a day [12,54,57]. These results are in alignment with the current study, where the majority of the study participants changed their pads twice a day. Studies in other parts of Pakistan have documented that the majority of girls clean their genitals with water only [12,36]. However, the current study revealed more encouraging findings, where almost equal numbers of girls used water and soap to clean their genitals. This could be attributed to the fact that a few participants from high schools received awareness sessions on menstrual hygiene from healthcare workers, so they might have learnt this practice from them. This study also investigated the bathing practices of young girls during menstruation, and a major proportion of the girls (59.3%) bathed/washed their bodies when their periods were over. This may be because of the social stigma attached to menstruation, the cold weather of Gilgit, and the unavailability of warm water. These findings are supported by a few studies conducted in Pakistan and other countries [12,60]; however, a systematic review of LMICs concluded that girls from Turkey, Nigeria, Kenya, Iran, and Egypt bathed daily [5].

The present study also found that the majority of the participants avoided working outside the home and avoided taking a bath or washing the body when menstruating, while a few participants (19.8%) avoided exercise. This may be due to the restrictions of society, related to the myth that, during menstruation, girls are more vulnerable to evil spirits and disease, and they can bring embarrassment to the family if blood stains are found on their clothes. These findings are also reflected in studies from Pakistan [47,60], India [61], Nepal [62], and Saudi Arabia [63].

### 4.4. Strengths, Limitations, and Future Research

The findings of this study have provided baseline data in the context of Gilgit about the knowledge, attitudes, and practices regarding menstrual hygiene, which can be used for future studies; to the best of the researchers’ knowledge, this is the first study that has been conducted in Gilgit, Pakistan. However, one of the limitations is that, due to time constraints and a limited budget, this study was conducted only in one district of Gilgit.

The findings of this study can be used to assess the KAP of young girls who do not attend school or who are studying in private institutions. Furthermore, an interventional study should be conducted to assess the results of educational programs on knowledge, attitudes, and practices regarding menstrual hygiene. Nurses and female health workers could play a central role in providing up-to-date and reliable information through outreach programs. This could contribute to improving the sexual and reproductive health of adolescents and young girls, as well as the whole community.

## 5. Conclusions

This study concludes that menstrual hygiene is a great challenge for young girls residing in rural areas. The study findings show that girls have a poor level of knowledge, attitudes, and practices regarding menstrual hygiene. The health sector and school authorities should play a greater role in creating awareness about menstrual hygiene.

## Figures and Tables

**Table 1:** Demographic characteristics of the study participants (n = 300).

Variable	Frequency (n)	Percentage (%)
Age in years (mean ± SD)	17.55 ± 1.72	42
Class of study		
7th–10th grade	95	31.70%
11th–12th grade	204	68.20%
Elder sister		
Yes	212	70.30%
No	88	29.30%
Father’s education		
No education	99	33%
Primary education	13	4.30%
Middle education	42	14%
Secondary education	61	20.30%
Intermediate	50	16.70%
Bachelor’s and above	36	12%
Mother’s education		
No education	196	65.30%
Primary education	20	6.70%
Middle education	37	12.30%
Secondary education	30	10%
Intermediate	13	4.30%
Bachelor’s and above	4	1.30%
Toilet facility at		
home	300	100%
Toilet facility at school/college	300	100%
Soap for washing at home	300	100%
Soap for washing at school/college		
Yes	209	69.70%
No	91	30.30%
Facilities at home		
TV	266	56.10%
Radio	64	13.50%
Internet	144	30.40%
First heard about menstruation		
˂10 years	12	4%
10–12 years	99	33%
12–15 years	154	51.30%
˃15 years	35	11.70%
Age at menarche		
Before 11 years	5	1.70%
12–14 years	140	46.70%
15–16 years	134	44.40%
Over 16 years	21	7%

**Table 2:** Knowledge of young girls regarding menstrual hygiene (n = 300).

Variable	Frequency (n)	Percentage (%)
Discussed first periods		
Mother	145	48.30%
Sister	112	37.30%
Grandmother	1	0.30%
Friend	30	10%
No one	12	4%
At first period, were you already aware of it?		
Yes	141	47%
No	155	51.70%
Do not remember	4	1.30%
If yes, source of information		
I was informed directly by teacher	6	4.30%
I was informed directly by mother	59	41.80%
I was informed directly by sister	45	31.60%
I was informed directly by friends/peers	20	14.20%
I indirectly came to know from mother	4	2.80%
I indirectly came to know from sister	7	5%
Where does the menstrual blood come from?		
Uterus	97	32.30%
Abdomen	26	8.70%
I don’t know	177	59%
Who experiences menstruation?		
Ill and injured women	4	1.30%
Women of reproductive age	137	45.70%
Adolescent girls only	99	33.30%
Women who have had children	3	1%
Do not know	57	19%
Menstruation is		
Natural process	276	96.50%
Disease	7	2.40%
Secret	1	0.30%
Do not know	16	0.70%
Menstruation is a sign of		
Something has gone wrong	11	3.60%
Being ready to marry	65	21.20%
Fertility	89	29%
Uncleanliness	87	28.30%
Do not know	55	17.90%
When does menstruation usually start?		
When someone is sexually active	22	7.30%
During adolescence	207	68.80%
Do not know	72	23.90%

**Table 3:** Attitudes of young girls regarding menstrual hygiene (n = 300).

Variable	Frequency (n = 300)	Percentage (%)
Activities to be avoided during menstruation		
Going to school	28	7.50%
Working outside home	57	15.20%
Washing the body/bathing	141	37.60%
Cooking/housekeeping	19	5.10%
Touching stored food/utensils	7	1.90%
Talking to boys	15	4%
Exercise	64	17.10%
None of these	44	11.70%
Talk about menstruation comfortably		
No one	35	9.70%
Teacher	2	0.60%
Mother	137	38.20%
Sister	7	29%
Other family members/relatives	1	0.30%
Friends/female peers	33	9.20%
Health workers	47	13.10%
What kinds of problems do girls face at menarche?		
Feel anxiety and stress	202	67.30%
She thinks she has a medical problem	17	5.70%
She is unaware of what to do	34	11.30%
She does not share with anyone	27	9%
She does not face any problems	20	6.70%
Girls should be made aware before first cycle		
Yes	273	91%
No	20	6.70%
Do not know	7	2.30%
If yes, who should tell her?		
Mother, sister, friend, relatives, or peers	238	42.80%
Schoolteacher	6	1.10%
A health worker	39	7%

**Table 4:** Practices of young girls regarding menstrual hygiene (n = 300).

Variable	Frequency (n)	Percentage (%)
Absorbent usually used during menstruation		
New cloth	34	11.30%
Disposable pads (available in market)	92	30.70%
Tissue/toilet roll	18	6%
Cotton	13	4.30%
Reusable cloth/towel	143	47.70%
What do you do with the used disposable material (disposable material users only)?		
Get rid of it in a field/bush	1	0.30%
Put it into the latrine	22	7.30%
Use waste bin	19	6.30%
Burn them	74	24.70%
Other (bury in the land)	8	2.70%
What do you do with the used reusable material (reusable material users only)?		
I wash it with water and soap/detergent and keep it for next use	145	48.30%
I wash it only with water	25	1.70%
I do not wash	1	0.30%
Dry the washed absorbent (reusable material users only)		
In the sun outside	68	39.50%
In the shade outside	18	10.50%
Hide under other clothes	50	29.10%
In the room inside	35	20.30%
How often do you change the absorbent?		
Once a day	69	23%
Twice a day	148	49.30%
Three times or more a day	82	27.30%
I do not change	1	0.30%
Materials used to clean genitals and area around genitals		
Water only	116	38.70%
Soap and water	114	38%
Plain paper/tissue	41	13.70%
Cloth	11	3.70%
Nothing	18	6%
How often do you bathe/wash your body during your period?		
Daily	12	4%
Every second day	52	17.30%
Every third day	58	19.30%
When period is finished	178	59.30%
Activities avoided during menstruation		
Going to school	31	8.40%
Working outside home	74	20.10%
Washing body	105	28.50%
Cooking/housekeeping	32	8.70%
Touching stored food/utensils	10	2.70%
Talking to boys	10	2.70%
Exercise	73	19.80%
None of these	33	9%

## Data Availability

The data that support the findings of this study are available from the corresponding author upon reasonable request.
